# Effects of Short-Term Feeding with Diets Containing Insect Meal on the Gut Microbiota of African Catfish Hybrids

**DOI:** 10.3390/ani15091338

**Published:** 2025-05-06

**Authors:** Balázs Libisch, Zsuzsanna J. Sándor, Tibor Keresztény, Chioma Lilian Ozoaduche, Péter P. Papp, Katalin Posta, Janka Biró, Viktor Stojkov, Vojislav Banjac, Nóra Adányi, Mária Berki, Éva Lengyel-Kónya, Rita Tömösközi-Farkas, Ferenc Olasz

**Affiliations:** 1 Agribiotechnology and Precision Breeding for Food Security National Laboratory, Institute of Genetics and Biotechnology, Hungarian University of Agriculture and Life Sciences, 2100 Gödöllő, Hungary; kereszteny.tibor@uni-mate.hu (T.K.); or chiomalilian01@gmail.com (C.L.O.); pppeter507@gmail.com (P.P.P.); posta.katalin@uni-mate.hu (K.P.); olasz.ferenc.gyorgy@uni-mate.hu (F.O.); 2 Research Center for Fisheries and Aquaculture, Institute of Aquaculture and Environmental Safety, Hungarian University of Agriculture and Life Sciences, 5540 Szarvas, Hungary; nagyne.biro.janka@uni-mate.hu; 3 Doctoral School of Biology, Hungarian University of Agriculture and Life Sciences, 2100 Gödöllő, Hungary; 4 Sustainable Environment Development Initiative (SEDI), Benin City 300102, Nigeria; 5 Institute of Food Technology, University of Novi Sad, 21000 Novi Sad, Serbia; viktor.stojkov@fins.uns.ac.rs (V.S.); vojislav.banjac@fins.uns.ac.rs (V.B.); 6 Institute of Food Science and Technology, Hungarian University of Agriculture and Life Sciences, 1118 Budapest, Hungary; nora.adanyi@gmail.com (N.A.); berki.maria@uni-mate.hu (M.B.); lengyelne.konya.eva@uni-mate.hu (É.L.-K.); tomoskozine.farkas.rita.adel@uni-mate.hu (R.T.-F.)

**Keywords:** African catfish, insect meal, intestinal microbiota, *Bacillus*, chitinase, antibiotic resistance

## Abstract

The impact of short-term feeding of three distinct diets containing insect meals on the intestinal microbiota of African catfish hybrid (*Clarias gariepinus × Heterobranchus longifilis*) juveniles was examined. The animals received experimental diets containing 30% insect meals derived from black soldier-fly larvae (BSL) (*Hermetia illucens*), yellow mealworm (*Tenebrio molitor*) or blue bottle-fly larvae (*Calliphora vicina*) for 18 days. The relative abundance of the *Bacillaceae*, the *Planococcaceae* and other bacteria significantly increased (*p* < 0.05) in the intestinal microbiota of the BSL group and also in the pooled group of the three catfish groups that received insect meals. Several strains of the *Bacillales* cultured from BSL feed had higher (*p* < 0.05) abundance in the intestinal microbiota of the BSL group compared to those of the control group. Among these *Bacillales* strains, a single *fosB* antibiotic resistance gene was identified. In the gut resistomes of both the BSL and the control catfish groups, the *tetA(P)*, *tetB(P)* and *lnu(C)* antibiotic-resistance determinants were detected, while *fosB* was detected only in the BSL group. Overall, the study showed that a short-term shift to diets containing insect meals can induce significant (q < 0.05) changes in the gut microbiota of the African catfish without the development of reduced α-diversity and without the overgrowth of bacteria pathogenic to fish.

## Simple Summary

African catfish is widely propagated in Africa, where Nigeria is the largest producer; in Europe, this species comprised over two-thirds of all EU catfish production in 2020, with Hungary as the biggest producer. The impact of short-term feeding of three distinct diets containing insect meal on the intestinal microflora of African catfish juveniles was examined to test whether a short-term dietary change could alter the composition of the fishes’ gut bacterial community. The fish received experimental diets containing 30% insect meal derived from black soldier-fly larvae, yellow mealworm or blue bottle-fly larvae for 18 days. The abundance of certain Gram-positive bacteria increased in the intestinal microflora of the catfish group receiving meal derived from black soldier-fly larvae and also in the pooled catfish group, which consisted of all fish that were fed insect meals. Several bacterial strains cultured from the feed containing meal derived from black soldier-fly larvae were also detected in the gut microflora of fish fed this diet. Overall, the study showed that a short-term shift to diets containing insect meals can cause significant changes in the bacterial composition of the African catfish intestinal microflora. These changes were associated neither with reduced bacterial diversity, nor with the overgrowth of bacteria pathogenic to fish.

## 1. Introduction

The ecological footprint of insect breeding is much lower than that of field crops, which make up the greatest share of animal feed. Insects develop and reproduce rapidly, can be kept on bio-waste and have high feed-utilization rates due to varying body temperatures: about two kilograms of waste are used to produce one kilogram of insect biomass [1,2]. Due to the limited availability of marine raw materials for fish-feed production, insect proteins may become increasingly necessary as feed ingredients in the future [3,4,5]. Animal protein derived from black soldier-fly larvae (BSL) (*Hermetia illucens*) and yellow mealworm (MW) (*Tenebrio molitor*) that are intended for the production of feed for farmed animals (other than fur animals) have already been approved in the European Union (EU) by Commission Regulation 2017/893. Blue bottle fly (BBF) (*Calliphora vicina*), also referred to as meat fly, is commonly used as bait or as an ingredient in boilies for fishing or angling. BBF has been assessed as a potential source of fish feed in several studies [4,6,7]. However, it has not yet been directly used in the aquafeed industry. Research indicates that blue bottle-fly larvae should present a promising and chemically safe alternative protein source for aquaculture feeds, offering digestibility comparable or superior to those of other insect meals [6,7].

Fish in the family Clariidae have great economic importance as food fish, and species of the genus *Clarias* are known as fast-growing protein sources. In Africa, *C. gariepinus* is widely propagated, and in Europe, the Netherlands and Hungary also produce substantial quantities [8]. Hybrids between *C. gariepinus* and *Heterobranchus longifilis* were reported to have qualities that make them desirable for pond culture, including better taste and nutritional qualities compared to non-hybrids, tolerance to unfavorable environmental conditions, resistance to disease and high market demand [8,9]. In total 6849 tons of African catfish were produced in Europe in 2020; that amount represented over two-thirds of all EU catfish production. Hungary is the biggest producer of this catfish species in Europe, where its production rose from virtually zero to over 3000 tons in twenty years. African catfish accounts for about 94% of intensive industrial fish production in Hungary, where reasons for its popularity include its good growth and feed utilization, its fishbone-free meat and its good fileting yields [10,11,12].

In general, *C. gariepinus* is considered omnivorous in its natural habitats in Africa, as it feeds on a variety of plants and animals [13,14]. Insects (such as Diptera, Ephemeroptera, Hemiptera and Plecoptera) form a part of their natural diet; higher proportions of insects are usually eaten by the smallest fish to support their growth [13,14,15]. The availability of food is dynamic and seasonal throughout the year in tropical environments, and changes in the size of fish may also have an effect on the feed types actually consumed. Accordingly, the feeding habits of African catfish can change on a seasonal basis; the observed variations might also be attributed to seasonal activities such as spawning and/or migration [15,16,17].

In aquaculture, reasons for short-term changes in the feed types used can potentially include health issues in the farmed fish, environmental changes (for example, in water temperature and quality), fluctuations in the cost and availability of feed ingredients or sudden changes in the market demand for specific types of fish products such as fish with higher content of omega-3 fatty acids [18,19,20].

In the recent decades, researchers have proposed several approaches to improve the sustainability of aquaculture production, with specific attention also to the effects of climate change and its consequences worldwide. Due to the high sensitivity of fish microbiomes to various environmental effects and changes, it is important to evaluate these sustainability strategies and to predict their short-, medium- and long-term impacts on the microbiota of farmed fish [1,2,5].

The gut microbiota of certain fish species may change considerably even after the fish are fed a single meal [21,22], and it has been shown that feeding can have an effect on the fish intestinal microbiota over even a short time frame (i.e., hours) [23,24]. In general, the diet and lifestyle of the host may both contribute to temporal changes in bacterial relative abundances and potentially to day-to-day variability in the gut microbiota [25]. Despite the associated commercial and scientific relevance, the *C. gariepinus × H. longifilis* hybrid have so far received comparatively less research attention regarding the composition of its intestinal microbiota. A recent study investigated the intestinal microbiota of blue catfish (*Ictalurus furcatus*) and channel catfish (*I. punctatus*) of the family Ictaluridae and found that they were dominated by the phyla Firmicutes (Bacillota), Fusobacteriota and Proteobacteria [26,27]. Gericke and colleagues investigated the effects of xylanase and arabinoxylan oligosaccharides on the growth performance and gut microbial diversity of *C. gariepinus* and concluded that dietary xylanase supplementation could significantly impact its gut microbial communities [28].

The present work is a follow up study to a short-term digestibility trial with African catfish hybrid (*C. gariepinus × H. longifilis*) juveniles [4] that determined the apparent digestibility coefficients (ADCs) of different nutrients in experimental diets containing 30% defatted BSL, MW or BBF meals. Microbiological evaluation of these experimental feeds showed that the BSL feed contained two to three orders of magnitude higher numbers of culturable aerobic bacteria than the other tested diets did [4]. Based on these observations, the aims of the current study were (1) to investigate the effects of short-term feeding with diets containing 30% insect meals on the intestinal microbiota of African catfish hybrid juveniles and thereby to test their adaptability to short-term dietary changes; and (2) to assess the potential contribution of the abundant culturable microflora of the BSL diet to the catfish gut microbiota and to its acquired antibiotic resistance gene repertoire.

## 2. Materials and Methods

### 2.1. Fish Feeding and Rearing Conditions

The trial was conducted using African catfish hybrid (*C. gariepinus × H. longifilis*) juveniles. Fish management and feed formulations are thoroughly detailed in our previous paper, which follows a commonly applied protocol for digestibility experiments [4]. Shortly, a basic diet with a high fish-meal content was initially produced. Subsequently, experimental diets were formulated by mixing each type of tested insect meal with the mash of the basic diet in a ratio of 30:70 (see Supplementary Table S1). The tested insect meals were MW, BSL and BBF meals. The feeds were designed and produced at the Research Centre for Feed Technology, Quality and Safety, Institute of Food Technology, University of Novi Sad (Novi Sad, Serbia) by using a twin-screw extruder (Bühler BTSK-30, Bühler, Uzwil, Switzerland). The diets satisfied the nutrient requirements of African catfish juveniles, providing adequate levels of protein, fat, and gross energy. The main differences between the experimental diets with different insect meals were in crude fiber, acid-detergent fiber, and chitin content. Further details on the experimental diets and their compositions are available in Supplementary Table S1.

The animal experiments and related samplings were approved by the Ethical Committee of the Research Center for Fisheries and Aquaculture, Institute of Aquaculture and Environmental Safety, Hungarian University of Agriculture and Life Sciences, under license no. BE/25/4302-3/2017. The Ethical Committee was established according to Hungarian State law 9/1999 (I. 27.), and it is operated according to the relevant Hungarian legislation concerning animal experiments, transportation of animals, and their welfare (40/2013. II. 14) [4]. The fish were anesthetized with Norcaicum/Tonogen-based anesthesia before harvesting [29].

Nine hundred African catfish juveniles with an average weight of 217.4 ± 9.5 g and originating from the institutional hatchery facility of the Research Center for Fisheries and Aquaculture (Szarvas, Hungary) were reared in a recirculation aquaculture system equipped with 1 m^3^ fiberglass tanks using Claria Float grower feed (Aller Aqua, Christiansfeld, Denmark). The three experimental groups (BSL, MW, BBF) and the control group (CONT) were established and randomly distributed in tank triplicates (75 fish/tank). After seven days of acclimatization, the fish were fed the experimental diets for 18 days. During the feeding period, the fish were hand-fed to apparent satiation three times per day. The water flow was adjusted to an average of 4.5 L/min per tank; the daily water change was 10%; the dissolved oxygen level was kept above 80% of saturation; ammonia-N was below 0.1 mL/L and the pH was in the range 7.8–8.4. Water temperature was set to 23 ± 1 °C.

### 2.2. Intestinal Sampling

Before the start of feeding with experimental diets, the intestinal contents of six animals were sampled to analyze their microbiota (START group, Supplementary Table S2). After 18 days of feeding with the experimental diets, six to nine fish from each treatment group were collected. The feces collection was conducted 6 h after the last feeding. Sampling lasted for up to 30 min, and the feeding schedule was adjusted accordingly to accommodate this timing. The animals were weighed, and their full lengths were measured (Supplementary Table S2). The body-weight and body-length data showed normal distributions and did not differ significantly between the treatment groups BBF, BSL and MW and CONT according to one-way ANOVA (*p* > 0.05). Intestinal contents were collected from the distal part of the intestine, from the section preceding the anus, into sterile plastic containers using sterile surgical instruments (scalpels, forceps and scissors). The collected samples were transported to the laboratory on ice under anaerobic conditions [30,31].

### 2.3. DNA Purification and Amplicon Sequencing

gDNA was purified from the intestinal-content samples using the AquaGenomic Kit (MultiTarget Pharmaceuticals, Salt Lake City, USA). DNA was amplified with tagged primers covering the V3–V4 region of the bacterial 16S rRNA gene [32], using the primers 16S-F (5′-TCGTCGGCAGCGTCAGATGTGTATAAGAGACAGCCTACGGGNGGCWGCAG) and 16S-R (5′-GTCTCGTGGGCTCGGAGATGTGTATAAGAGACAGGACTACHVGGTATCTAATCC). Polymerase chain reactions (PCR) and DNA purifications were performed according to Illumina’s demonstrated protocol (Part # 15044223 Rev. B; Illumina, San Diego, CA, USA). Briefly, 50 ng template DNA was used for target amplification with KAPA HiFi Hot Start Ready Mix (Roche, Basel, Switzerland) in a 25 cycle PCR1 reaction under the following amplification conditions: 95 °C for 3 min; 30 cycles (95 °C for 30 s, 55 °C for 30 s, 72 °C for 30 s); 72 °C for 5 min and 4 °C. The PCR products were purified with 0.8 volume KAPA Pure Beads (Roche, Basel, Switzerland). 10 ng of PCR1 products was used as template in index PCRs. The indexing was performed with Nextera XT index v2 Kit (Illumina, Santa Clara, CA, USA) primers with KAPA HiFi Hot Start Ready Mix in an 8 cycle PCR reaction: 95 °C for 3 min; 10 cycles (95 °C for 30 s, 55 °C for 30 s, 72 °C for 30 s); 72 °C for 5 min; 4 °C. The PCR products were purified with 0.8 volume KAPA Pure Beads. The libraries were quantified with the Equalbit 1x dsDNA HS Assay Kit (Vazyme Biotech, Nanjing, China) on an Infinite Pro 200 F Nano+ Fluorescent Plate Reader (Tecan, Männedorf, Switzerland). Amplicon sequencing of the V3-V4 regions of the 16S rRNA gene on Illumina MiSeq platform (Santa Clara, CA, USA) was performed by Xenovea Ltd. (Szeged, Hungary) for the 36 African catfish intestinal samples listed in Supplementary Table S2. The Galaxy FastQC tool [33] was applied to verify the quality and quantity of the raw amplicon sequencing data. The Illumina 2 × 300 bp paired-end sequencing reads were quality-filtered and merged by the PEAR Galaxy tool [34]. In total, 89,293–198,783 merged reads with mean per-base quality scores > Q30 were obtained and analyzed for each intestinal sample.

### 2.4. Analyses of the Intestinal Microbiota Composition

The bacterial compositions of the intestinal microbiota samples were analyzed using the Nephele QIIME1 16S Amplicon pipeline [35]. The 16S rRNA gene V3–V4 amplicon sequencing data obtained on the Illumina MiSeq platform were further quality-filtered at a Phred quality score of 19, and chimeras were identified and removed by UCHIME. V3–V4 amplicon sequences were clustered at the 97% identity level to operational taxonomic units (OTUs) by the open-reference method and were classified using the full-length SILVA Small Subunit rRNA reference database [36]. The OTU tables were normalized by the rarefaction method at a sampling depth of 89,293, and OTU tables were filtered by QIIME1 to remove OTUs below five counts. Shannon and Simpson α-diversity indices were calculated using the Nephele QIIME1 pipeline [35]. β-diversity analyses were performed using QIIME1 based on the Bray–Curtis distance matrix. The statistical significance of the results of the β-diversity analysis was tested by permutational multivariate analysis of variance (PERMANOVA), where PERMANOVA also calculates a q-value, an adjusted *p*-value for multiple-groups testing [35,37,38]. Differential abundance testing between treatment groups was performed by the non-parametric Kruskal–Wallis test with *p*-values adjusted by the Bonferroni correction for multiple comparisons using IBM SPSS Statistics 29.0 software (SPSS Inc., Chicago, IL, USA), where a difference was considered significant at *p* < 0.05. The compositions of the intestinal bacterial communities of the START and CONT groups were compared using the Wilcoxon Signed-Rank test [39,40]. Besides the individual treatment groups described in Section 2.2, a pooled group designated group IM was also analyzed. The IM group was defined by pooling together all three catfish groups fed distinct diets containing insect meal (that is, by pooling groups BSL, MW and BBF; see Supplementary Table S2). Low-abundance OTUs were filtered out before differential abundance testing at a relative abundance threshold of >0.01% for at least 50% of the examined samples [41,42]. Plots of relative-abundance data were created in Past 4.08 software [43].

### 2.5. Culturing and Characterization of Bacterial Isolates from BSL Feed

For culture, 100 mg of BSL feed was mixed with 1 mL of 1 × phosphate-buffered saline (PBS) solution of pH 7.4, and 100 µL aliquots were spread onto brain–heart infusion agar (Biolab Zrt., Budapest, Hungary), and incubated at 35 °C for 72 h. Isolated colonies were identified by PCR amplification and Sanger sequencing of their 16S rRNA genes using the universal primers 27F 5′-AGAGTTTGATCCTGGCTCAG-3′ and 1492R 5′-GGTTACCTT GTTACGACTT-3′. Sequencing was performed by BIOMI Ltd. (Gödöllő, Hungary). The isolates were identified by BLASTN searches of their 16S rRNA gene sequences against the 16S ribosomal RNA (Bacteria and Archaea type strains) database of the NCBI RefSeq Targeted Loci Project.

### 2.6. Whole Genome Sequencing of the Isolates Cultured from BSL Feed

Selected bacterial isolates cultured from BSL feed were subjected to whole-genome sequencing (WGS) by Xenovea Ltd. (Szeged, Hungary) on the Illumina MiSeq platform using 2 × 250 bp paired-end reads. De novo contig-level assembly of sequencing data was performed by the SPAdes v3.15.0 assembler, and the contig-level assemblies were submitted to the National Center for Biotechnology Information (NCBI) Genomes database under project PRJNA1006294. The isolates were identified by BLASTN searches of their 16S rRNA gene sequences against the 16S ribosomal RNA (Bacteria and Archaea type strains) database of the NCBI RefSeq Targeted Loci Project and also by use of the KmerFinder 3.2 tool (https://cge.food.dtu.dk/services/KmerFinder/, last accessed on 24 April 2025); these two approaches yielded consistent results.

### 2.7. Shotgun Metagenomic Sequencing and Assembly

gDNA was purified from 0.18–0.2 g intestinal contents from catfish AH25 and AH31 from the BSL diet group and catfish AH43 and AH44 from the CONT group (Supplementary Table S2) using the QIAamp Fast DNA Stool Mini Kit (QIAGEN, Hilden, Germany). Shotgun metagenomic sequencing on the Illumina NovaSeq 6000 platform was performed by iBioscience Ltd. (Pécs, Hungary) and Xenovea Ltd. (Szeged, Hungary) with 2 × 150 bp paired-end reads. The most frequently observed mean per-sequence quality scores were >Q34 for both forward and reverse Illumina reads, with zero per-base ambiguous nucleotide (N) content, indicating high-quality base calling. Raw shotgun-sequencing reads were submitted to the NCBI Sequence Read Archive (SRA) database under accession PRJNA1042795. Metagenomic contig assembly was conducted with the MEGAHIT de novo assembler [44,45] by metaSPAdes [46] and PEAR [34]. Basic statistical analyses for shotgun metagenomic contigs were performed using the Fasta Statistics tool [47].

### 2.8. Functional Annotation of Shotgun Metagenomic and WGS Data

Shotgun metagenomic and WGS contigs were annotated with the Rapid Annotations using Subsystems Technology toolkit (RASTtk) [48]. Genes predicted to encode chitinase enzymes by RASTtk were further analyzed by BLASTX searches against the NCBI Protein database. Acquired antibiotic resistance genes (ARGs) were identified using the ABRicate tool [31,49], which screened query sequences against the ResFinder 4.1 database [50] with the settings ≥80% sequence identity (EFSA BIOHAZ, 2023) and ≥40% minimum coverage. Metagenomic contigs harboring ARGs were also annotated using Prokka [51] and BLASTP searches of translated ORFs against the NCBI Protein database. Schematic diagrams of annotated contigs were generated in SnapGene Viewer (Insightful Science, Woburn, MA, USA) [31].

### 2.9. Statistical Data Analyses

Statistical data analyses were performed using IBM SPSS Statistics 29.0 software (IBM SPSS Inc., Chicago, IL, USA). Testing for normality of data was carried out using the Shapiro–Wilk Test. Differential abundance testing between treatment groups was carried out using the non-parametric Kruskal–Wallis test with *p*-values adjusted by the Bonferroni correction for multiple comparisons. The α-diversity of the intestinal microbiota was assessed by the Shannon and Simpson indices, which were compared between treatment groups by the Kruskal–Wallis test using Bonferroni-adjusted *p*-values. The statistical significance of the results of β-diversity analyses was tested by PERMANOVA using the Beta-diversity group significance tool (Galaxy Version 2024.10.0+q2galaxy.2024.10.0), which is a non-parametric multivariate statistical permutation test [37]. PERMANOVA tests whether distances between samples within one treatment group (that is, within-group distances) differ from the distances to samples in another group (that is, across-group distances) [38]. Differences between the intestinal microbiota compositions of treatment groups in the β-diversity analyses were considered significant at q < 0.05.

## 3. Results

### 3.1. Bacterial Composition of the African Catfish Intestinal Microbiota

**Figure 1:**
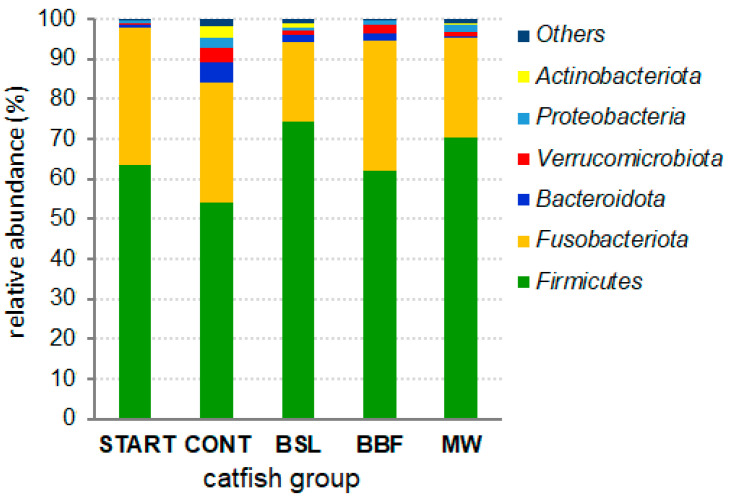
Phylum-level mean bacterial compositions of the intestinal microbiota.

The phylum-level mean bacterial compositions of the intestinal microbiota in the five catfish groups are summarized in Figure 1. The main phyla were the Firmicutes, Fusobacteriota, Bacteroidota, Verrucomicrobiota, Proteobacteria and Actinobacteriota, where the Firmicutes and Fusobacteriota together accounted for 84.1% (in the CONT group) to 97.8% (in the START group) of the intestinal bacteria.

**Figure 2:**
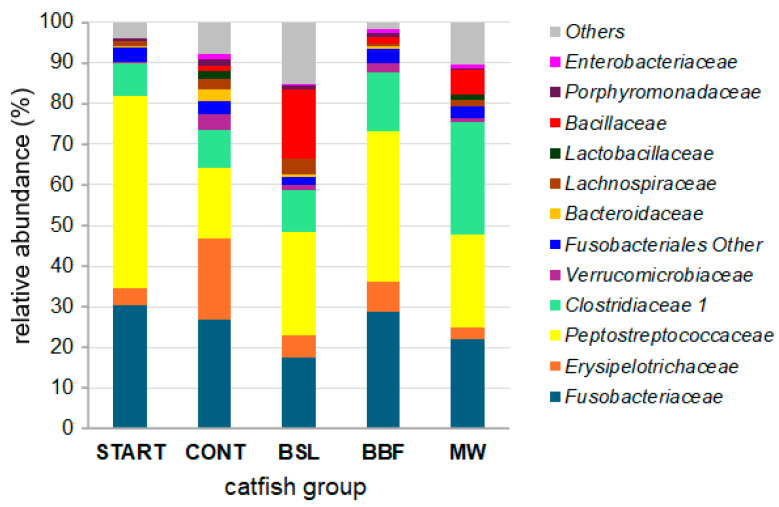
Family-level mean bacterial compositions of the intestinal microbiota.

The family-level mean bacterial compositions are shown in Figure 2. The main families in the control (CONT) group were the *Fusobacteriaceae*, *Erysipelotrichaceae*, *Peptostrepto-coccaceae*, *Clostridiaceae 1* and *Verrucomicrobiaceae*, while the main genera and their mean relative abundances in the CONT group were *Cetobacterium* (26.7%), *Turicibacter* (19.8%), *Romboutsia* (14.3%), *Clostridium sensu stricto 1* (5.6%), *Akkermansia* (3.7%), *Fusobacteriales, other* (3.08%) and *Bacteroides* (3.01%).

### 3.2. Changes in the Intestinal Microbiota Between Groups START and CONT

A significant increase in relative abundances of bacteria from the phylum Proteobacteria was observed (*p* = 0.046) between the START and CONT groups. The relative abundance of the family *Verrucomicrobiaceae* was also higher in the CONT group (*p* = 0.046), while that of the *Peptostreptococcaeae* decreased (*p* = 0.028). Genus-level significant changes (*p* < 0.05) are shown in Supplementary Figure S1.

### 3.3. Effects of Short-Term Feeding with Diets Containing Insect Meal on the Intestinal Microbiota

**Figure 3:**
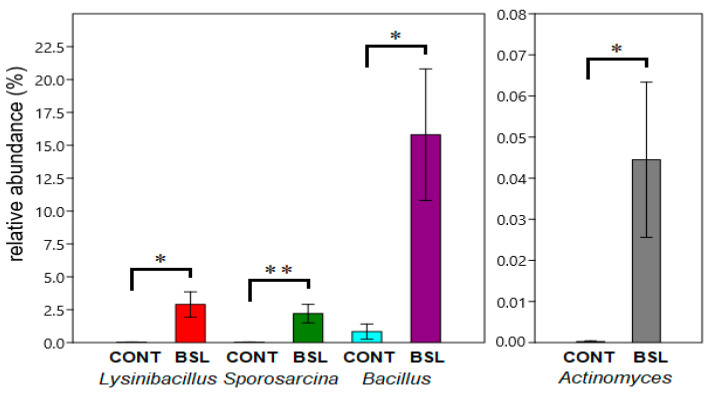
Significant differences in relative abundances (±SE) of the genera *Lysinibacillus* (*p* = 0.013), *Sporosarcina* (*p* = 0.007), *Bacillus* (*p* = 0.023) and *Actinomyces* (*p* = 0.037) between groups CONT and BSL. * Difference significant at the *p* < 0.05 level. ** Difference significant at the *p* < 0.01 level.

**Figure 4:**
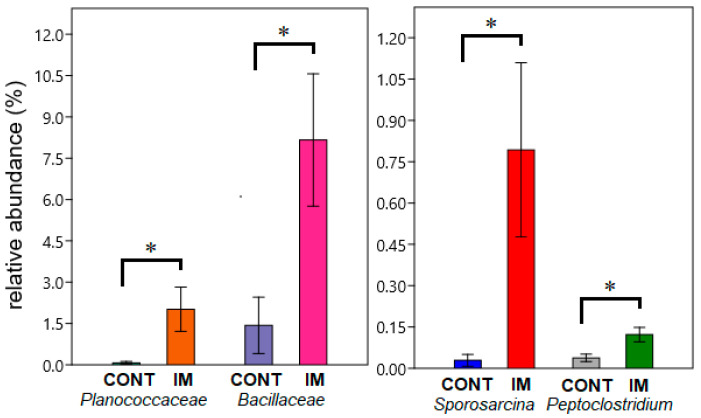
Significant differences in relative abundances (±SE) of the families *Planococcaceae* (*p* = 0.034) and *Bacillaceae* (*p* = 0.040) and of the genera *Sporosarcina* (*p* = 0.022) and *Peptoclostridium* (*p* = 0.044) between groups CONT and IM. * Difference significant at the *p* < 0.05 level.

Significant changes in the relative abundances of various bacterial taxa in response to experimental diets are shown in Figure 3 and Figure 4. Differential abundance testing of intestinal bacteria between treatment groups also involved the comparison of group CONT with the three insect-meal groups BSL, BBF and MW as one pooled group (designated group IM, Supplementary Table S2).

Relative abundances of the genera *Bacillus*, *Sporosarcina*, *Lysinibacillus* and *Actinomyces* were higher in the BSL group compared to the control group; higher abundances of the *Bacillaceae* (*p* = 0.031), *Planococcaceae* (*p* = 0.008) and the Bacillales (*p* = 0.041) were also observed. The phylum Actinobacteriota was elevated in the BSL group compared to the BBF group, at 1.11% vs. 0.077% (*p* = 0.022). The bacterial composition of the pooled IM group also showed significant changes compared to the control group. The families *Bacillaceae* (*p* = 0.040) and *Planococcaceae* (*p* = 0.034) displayed higher relative abundance in group IM compared to CONT, as did the genera *Sporosarcina* (*p* = 0.022) and *Peptoclostridium* (*p* = 0.044) (Figure 4).

Moreover, significant positive correlations were found by Pearson analyses between the chitin contents of the experimental diets and the relative abundances of the taxa *Bacillus* (r = 0.55, *p* < 0.01), *Sporosarcina* (r = 0.55, *p* < 0.01), *Lysinibacillus* (r = 0.54, *p* < 0.01), *Bacillaceae* (r = 0.55, *p* < 0.01), and *Planococcaceae* (r = 0.55, *p* < 0.01) in the intestine.

### 3.4. α-Diversity Analyses of the Intestinal Microbiota

**Figure 5:**
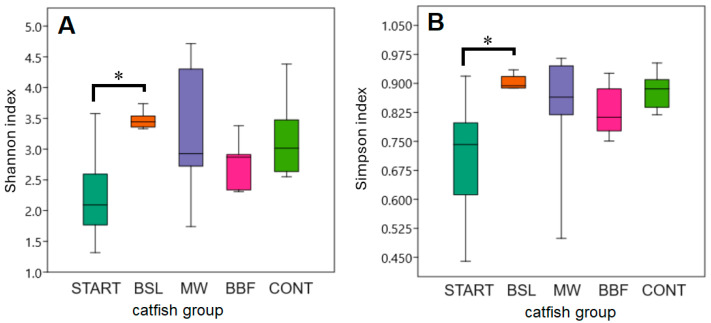
Shannon (**A**) and Simpson (**B**) α-diversity indices of the catfish intestinal microbiota. * Differences significant at the *p* < 0.05 level.

Both the Shannon and the Simpson indices [39,40] showed significant (*p* < 0.05) increases between the START and the BSL groups, but significant differences were not found between the CONT and the BSL, MW and BBF groups (Figure 5). However, a trend toward higher α-diversity was found in all catfish groups compared to the START group.

### 3.5. β-Diversity Analyses of the Intestinal Microbiota

PERMANOVA was applied using the Bray–Curtis distance metrics and then pairwise comparisons between the treatment groups. The analysis was performed using 999 permutations and provided the associated pairwise *p*- and q-values.

**Figure 6:**
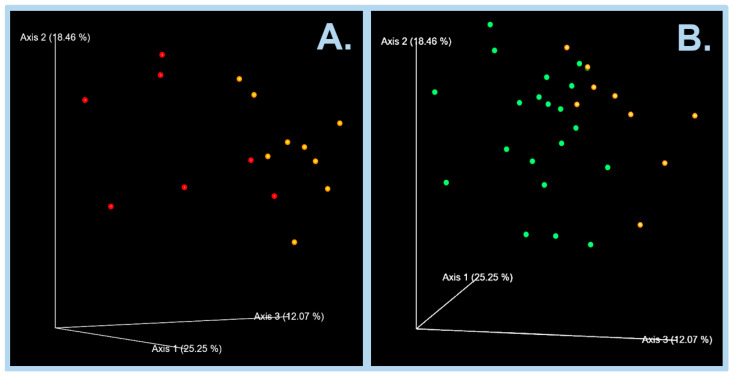
Principal coordinate analysis (PCoA) of Bray–Curtis distances for catfish groups CONT (orange points •) versus group BSL (red points •, (**A**)) or group IM (green points •, (**B**)). Each sample is represented by a colored point in a multidimensional space based on the composition of the bacterial population in each corresponding catfish intestinal sample.

There were significant differences (q < 0.05) among the compositions of the intestinal microbiota of the START, CONT, BSL and IM catfish groups (Table 1 and Figure 6), while in the other pairwise group comparisons, q values > 0.05 were obtained, with the pairwise *p*-values ranging between 0.023 and 0.36.

### 3.6. Identification of Bacterial Strains Cultured from BSL Feed

The BSL feed was subjected to additional culture-based studies because it contained two to three orders of magnitude higher numbers of mesophilic aerobic bacteria compared to the other feeds [4] and because the BSL diet induced the most pronounced changes in the composition of the catfish gut microbiota during a short-term feeding trial. Ten isolated colonies cultured on brain–heart infusion (BHI) agar plates from BSL feed were randomly selected for Sanger sequencing of their 16S rRNA genes (Table 2). Of these ten isolates, nine belonged to the Bacillales (phylum Firmicutes) and one to the Micrococcales (phylum Actinobacteriota).

**Figure 7:**
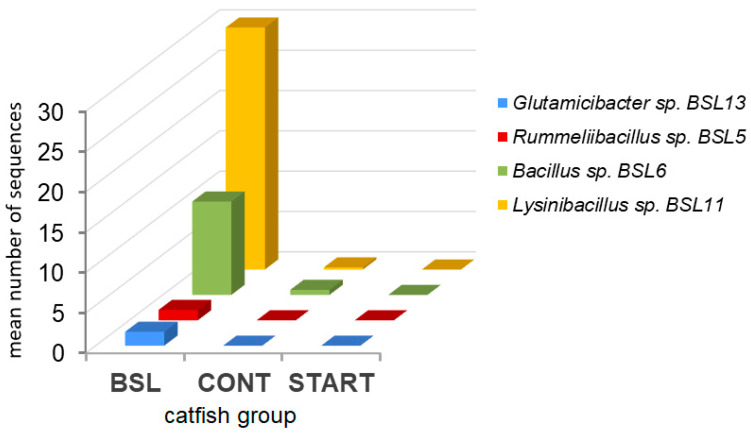
Mean number of 16S rRNA gene sequences of bacteria cultured from BSL feed detected in catfish intestinal amplicon-sequencing data.

The 16S rRNA genes of all cultured strains in Table 2 were detected with 100% identity in the intestinal 16S rRNA gene amplicon-sequencing data of the BSL catfish group (Figure 7). A Kruskal–Wallis test showed that the 16S rRNA gene sequences of the strains cultured from BSL feed were significantly more abundant in the BSL group compared to the CONT and START groups (*p* < 0.05, in pairwise comparisons); their copy number was zero in the START group for all strains (Figure 7).

### 3.7. Screening for Acquired Antibiotic-Resistance and Chitinase Genes

Bacterial isolates of each genus cultured from the BSL feed (Table 2) were submitted for further characterization by WGS; these were designated *Rummeliibacillus* sp. strain BSL5, *Bacillus* sp. strain BSL6, *Lysinibacillus* sp. strain BSL11 and *Glutamicibacter* sp. strain BSL13. Their WGS data were screened for acquired ARGs against the ResFinder 4.1 database [50]. One acquired ARG was detected, a single *fosB* fosfomycin thiol transferase gene in *Bacillus* sp. strain BSL6.

The acquired ARGs identified in the intestinal microbiomes of the BSL and CONT groups at ≥40% coverage and ≥80% identity with the reference genes are summarized in Table 3. ARGs included genes encoding resistance to tetracyclines, macrolides and other antibiotic classes; a *fosB* gene was also found. The *lnu(C), tetA(P)* and *tetB(P)* determinants were harbored by two intestinal metagenomic contigs of 6140 bp and 12,971 bp, respectively, together with genes encoding various *Cetobacterium* proteins, as well as IS*Sag10*, IS*91* and IS*4* family transposases (Supplementary Figure S2).

A variety of known and putative chitinase genes detected in bacterial strains cultured from BSL feed and in the intestinal microbiomes of the BSL and CONT groups are summarized in Supplementary Tables S3, S4 and S5, respectively. These data pointed to the presence of a diverse bacterial chitinase gene repertoire in the intestinal microbiomes of the BSL catfish group [52,53,54,55].

## 4. Discussion

The gut microbiota composition in fish varies with their diet and species, where diet was proposed to be a stronger driver [56]. Generally, the intestinal microbiota of most fish are composed of the phyla Firmicutes, Fusobacteriota, Actinobacteriota, Bacteroidota, and Proteobacteria cumulatively at higher than 80% proportion [57]. In a finding similar to results obtained in the current study (Figure 1), the Fusobacteriota and the Firmicutes were the two most abundant phyla in the gut-associated microbiota of ictalurid catfish, where the Fusobacteriota dominated the microbiota of blue catfish and the Firmicutes dominated in channel catfish strains [26]. At the genus level, the most abundant bacterial genera in the CONT group of our study were *Cetobacterium*, *Turicibacter*, *Romboutsia*, *Clostridium sensu stricto 1*, *Akkermansia*, *Fusobacteriales, other* and *Bacteroides.* For comparison, *Cetobacterium*, *Turicibacter*, *Romboutsia*, *Clostridium sensu stricto 1* and *Bacteroides* were also within the most abundant genera in the gut-associated microbiota of *C. gariepinus* full siblings in the control group of a trial examining the effects of *Arthrospira platensis* on the microbiomes of African catfish [58].

In the BSL catfish group, a statistically significant increase was observed in the relative abundances of the genera *Bacillus*, *Lysinibacillus*, *Sporosarcina* and *Actinomyces*, where the relative abundance of the genus *Bacillus* was 19-fold higher in the BSL group compared to that in the control group (Figure 3). Of these genera, *Sporosarcina* was recorded as the genus with the second-highest abundance in the microbiota in BSL production residue, and it was isolated from black soldier-fly eggs [59,60]. In addition, *Sporosarcina* sp. became the most abundant bacterial genus in the microbial communities in the finfish substrate after BSL were grown in the laboratory on finfish seafood waste for 14 days [61]. *Lysinibacillus* was also isolated from black soldier-fly eggs, and *Lysinibacillus* was enriched in the intestinal microbiota of Atlantic salmon (*Salmo salar*) fed an insect-meal diet in which BSL meal composed 60% of feed ingredients [60,62].

Chitin was present in the highest proportion in the BSL feed (4.91 ± 0.54%) (Supplementary Table S1), and it may act as a substrate that increases the proliferation of chitinolytic bacteria within insect meals and/or feeds containing insect meal. Chitinolytic bacteria are mainly represented by the Firmicutes and include many *Bacillus* species [63]. *Bacillus* spp. were identified in the intestine of Atlantic salmon fed a diet with 5% chitin, and these bacteria also showed the highest chitinase activity in vitro [64]. The *Actinomyces*, the *Bacillaceae* and *Bacillus* were enriched (among some other taxa) in both the intestinal digesta and the intestinal mucosa of Atlantic salmon fed a diet containing about 15% BSL meal [65].

An elevated abundance of *Bacillaceae* was also reported in a previous study of rainbow trout (*Oncorhynchus mykiss*) fed larval and prepupal stages of black soldier-fly meal [66]. In another study conducted on rainbow trout, an increase in the *Bacillus* genus was detected in response to an experimental diet containing 15% BSL meal to replace a control diet containing 50% fish meal [67]. A higher abundance of bacteria assigned to the family *Planococcaceae* was found in the gut of rainbow trout fed 20% and 30% insect meal from *H. illucens* prepupae in their diet [68]. In alignment with these observations, a significant increase in representation of the families *Bacillaceae* and *Planococcaceae* was detected in our study in the BSL-fed African catfish group and also in the pooled group of the three treatment groups fed insect meals (group IM, Figure 4); an increase in bacteria from the *Bacillales* [67,68] was also observed in the BSL catfish group.

The β-diversity analyses confirmed that an 18-day period of feeding diets containing insect meals significantly (q < 0.05) altered the compositions of intestinal microbiota of catfish groups BSL and IM compared to the control group (Table 1 and Figure 6).

Significantly higher Shannon and Simpson indices of the gut microbiota were found in the BSL group compared to the START group (*p* < 0.05), and a non-significant increase was also found in the CONT, BBF and MW groups; by contrast, the Shannon and Simpson indices of the CONT versus BSL, BBF and MW groups did not differ significantly (Figure 5). Therefore, dysbiosis in the intestinal microbiota, as indicated by reduced α-diversity [69], was not observed in response to short-term dietary insect-meal exposure. Instead, a more complex intestinal microbiota was established in all dietary groups compared to the START group (Figure 5). Likewise, a substantially longer 10-week feeding period with 50% or 100% replacement of fish meal with cricket meal did not cause significant changes to the Shannon and Simpson α-diversity indices compared to the control group in the intestinal microbiota of juvenile channel catfish [70].

The mean Shannon index of the *C. gariepinus* intestinal microbiota increased from 1.63 to 2.78 between 3 and 6 months of ages, respectively, in the control group of a study testing two feed additives [28]. Trends of increasing α-diversity were also reported during the development of Southern catfish (*Silurus meridionalis*), where the mean Shannon index of the intestinal microbiota rose from 1.81 to 2.75 between 8 and 125 days of age [71]. The Shannon diversity of the gut microbiota increased over time for aquaculture-cultivated African catfish kept in pools with a closed water supply [72]. These and other, similar findings indicate that direct effects associated with host age might also play a role in shaping gut microbial diversity in African catfish [71,73,74].

Microbiological evaluation of the experimental feeds applied in this study revealed that the average numbers of colony forming units (CFUs) of mesophilic aerobic bacteria in the CONT, BBF and MW feeds were about 10^2^–10^3^ CFU/g, while the BSL feed contained two to three orders of magnitude more CFUs than the other tested feeds [4]. Furthermore, the most pronounced changes were observed in the gut microbiota of the BSL group in response to short-term insect meal feeding, where both the Shannon and Simpson indices rose significantly (*p* < 0.05) compared to the START group (Figure 3 and Figure 5). To assess potential interactions between the microflora of the BSL feed and the intestinal microbiota of the BSL catfish group, additional culture-based and metagenomics analyses were performed (Table 2 and Table 3, Supplementary Tables S3–S5, and Figure 7). Several strains of bacteria enriched in the intestines of the BSL group were cultured from the BSL feed (Table 2). Moreover, a significantly higher number of 16S rRNA gene sequences of bacteria cultured from BSL feed (*p* < 0.05) were identified in the intestinal microbiomes of the BSL catfish group compared to the control (Figure 7). These data indicate that the microflora of the BSL feed also contributed to the gut microbiota of the BSL catfish group during this short-term feeding trial. Likewise, it was shown that diet likely served as an inoculum to the channel catfish gut because of a parallel pattern in the abundance of *Streptococcus* between the channel catfish gut microbiota and the microbiota of their diet [75]. Differential colonization by food-associated microbes between fish populations has also been described in stickleback gut microbiota [76].

The ADCs for chitin determined in the same short-term digestibility trial [4] showed that African catfish was able to digest chitin from the tested insect meals in different ratios and with ADCs comparable to those obtained in tilapia [77]. A gastric chitinase mRNA was detected at 3 days post-hatch and was highly expressed from 4 days post-hatch onwards in larvae of African catfish (*C. gariepinus*) during early development [78]. However, the endogenous chitinolytic activity did not show any discernible trend with increased inclusion of mopane worms, and increased endogenous chitinase activity did not result in a higher specific growth rate of African catfish [79]. Based on these findings, the African catfish gut microbiota might play a significant role in the utilization of dietary chitin through bacterial enzymes associated with chitin degradation [53].

In accordance with the observations of the current study (Figure 3), an increase in the relative abundances of *Actinomyces* and *Bacillus* was found when Atlantic salmon were fed fly larvae [80] or a diet containing about 15% BSL meal [65]. As both *Actinomyces* and *Bacillus* are potential chitin degraders, it was proposed that the activity of bacterial chitinases might have reduced the level of chitin substrates available to endogenous fish chitinases in the gut [80]. Further analyses identified several known or putative chitinase genes in the intestinal metagenome of the BSL catfish group (Supplementary Table S4) and in bacterial isolates cultured from BSL feed (Supplementary Table S3), pointing to the presence of a gut bacterial community with potential for chitin degradation [52,53,54,55]. Accordingly, the chitin digestibility for the BSL meal used in our study was also high (96.05%) [4], and the number of chitinase gene types detected was markedly lower in the CONT group compared to the BSL group (Supplementary Table S5).

The positive Pearson correlation (*p* < 0.01, Section 3.3) found between the chitin content of the experimental diets and the relative abundances of *Bacillus*, *Bacillaceae* and other taxa indicates that dietary chitin might increase the proliferation of chitinolytic intestinal bacteria and that an increased amount of *Bacillus* might also represent an effect of dietary chitin. The *Bacillus* genus is one of the most commonly used probiotics in aquaculture; it is applied to enhance fish immune responses and disease resistance [67]. Concerning the *Rummeliibacillus* strain BSL5 cultured from BSL feed (Table 2), it is notable that the *Rummeliibacillus* genus has been considered as a potential source of strains for use as feed additives to enhance the growth, health status and intestinal microbiota in fish and poultry [81,82,83]. The absence of acquired ARGs in the genome of *Rummeliibacillus* strain BSL5 means that this strain meets the requirement set by the European Food Safety Authority (EFSA) for the development of microorganisms intended for use in the food or feed chains [84].

Metagenomic analyses revealed an intestinal acquired resistome in the CONT and BSL groups that mainly consisted of ARGs that have been identified in African catfish or channel catfish in other countries as well (Table 3, Supplementary Table S6) [85,86,87,88,89,90,91,92,93,94,95]. Comparison with ARGs detected in the intestines of common carp from the same geographical region in Hungary [31] showed that the tetracycline class was present in both species and that a *sul1* and a *qnr*-type acquired quinolone-resistance determinant (*qnrS2* or *qnrD1*, respectively) was also detected in both fishes. The *sul1* dihydropteroate synthase gene takes part in forming the 3′-conserved sequence (3′-CS) of class 1 integrons, which are prevalent in antibiotic-resistant strains of *Aeromonas* spp., *Pseudomonas* spp. and other genera widely distributed in aquatic environments [96,97,98]. Overall, of the ARGs identified in African catfish (Table 3), only the *sul1* determinant was shared with the resistome described from common carp in Hungary [31], implying that their distinct gut microflora were associated with different acquired resistome profiles. The *qnrD* determinant has already been reported in Hungary from *Enterobacteriaceae* isolates cultured from human urine clinical samples [99] and from the urine of a companion dog [100], but it has not yet been reported from aquaculture.

The *tetA(P)* and *tetB(P)* genes (Table 3) were harbored by the same metagenomic contig that carried several *Cetobacterium* genes in the immediate vicinity of *tetA(P)* and *tetB(P)*; similarly, the *lnu(C)* gene was also neighbored by *Cetobacterium* genes on another metagenomic contig (Supplementary Figure S2). These three acquired ARGs were therefore likely carried by intestinal isolates of *Cetobacterium* spp., a dominant genus of the family *Fusobacteriaceae* in the gut of African catfish juveniles (Figure 2). A *Cetobacterium* isolate featuring *tetA(P)*, *tetB(P)* and *lnu(C)* genes and highly adapted to fish gastrointestinal conditions has been reported from the gut microbiota of Nile tilapia [101]. Furthermore, the *tetA(P)*, *lnu(C)* and *sul1* genes (Table 3) have also been detected in untreated sewage wastewater in Hungary, where the North Budapest wastewater-treatment plant treats both communal wastewater and organic wastes of animal origin [31,102].

Considering the *fosB* gene identified in the *Bacillus* sp. BSL6 isolate, research for the Human Microbiome Project reference database revealed that of 133 FosB homologues, 89 homologues were distributed in the family *Bacillaceae* and that 61.7% of *fosB*-containing bacteria belonged to the genus *Bacillus* [103]. The *fosB* gene may thus be considered a widely distributed ARG among *Bacillus* strains in human-impacted environments. The *fosB* gene was detected only in the analyzed gut samples of the BSL catfish group (Table 3), which is a further supporting finding that the microflora of the BSL feed might have contributed to the gut microbiota of this catfish group.

Longer-term feeding trials using >30% insect meal in the tested diets showed that the growth of African catfish was not significantly reduced during the 4-week or 6-week feeding periods [104,105]. Likewise, replacement of up to 100% of fishmeal with cricket meal did not negatively influence the growth performance of juvenile channel catfish (*Ictalurus punctatus*) in the course of a 10-week feeding trial [70].

## 5. Conclusions

In the course of a short-term feeding trial, African catfish hybrid juveniles fed experimental feeds containing 30% BSL or BBF-type insect meals [4] were able to utilize these feeds well; accordingly, no characteristics of a dysbiotic intestinal microbiota, such as reduced α-diversity or the overgrowth of enteric bacteria with the potential to be pathogenic in fish, were observed. Additionally, the intestinal abundance of bacterial genera expected to be involved in the utilization of diets containing insect meals increased. Genomic and metagenomic analyses showed a contribution of the microflora of the BSL feed to the intestinal microbiota of the BSL catfish group, where the bacterial strains cultured from the BSL feed did not harbor acquired ARGs, with the exception of a single *fosB* gene. Overall, our observations indicate that the intestinal microbiota of catfish juveniles can change significantly (q < 0.05) and adapt in a relatively short period to a new type of diet containing 30% insect-derived raw materials. The most pronounced short-time changes in the catfish intestinal microbiota were associated with the BSL diet, which contained two to three orders of magnitude greater numbers of aerobic culturable bacteria compared to the other tested diets. The BSL diet also proved significantly more digestible for protein, fat, ash, and phosphorus than the control diet. To our knowledge, this is the first short-term feeding trial investigating the plasticity of the intestinal microbiota of farmed *C. gariepinus × H. longifilis* hybrids. Further studies are planned to characterize in vitro whether the bacterial strains cultured from BSL feed have a significant chitin-degradation potential and to assess their possible probiotic value in fish farming.

## Figures and Tables

**Table 1:** PERMANOVA analysis of Bray–Curtis dissimilarity between catfish groups.

Group 1	Group 2	Permutations	Pseudo-F	*p*-Value	q-Value
START	CONT	999	3.51	0.001	0.01
BSL	CONT	999	3.91	0.002	0.01
BSL	START	999	3.84	0.006	0.02
IM	CONT	999	2.70	0.013	0.019

**Table 2:** Identification of bacterial isolates cultured from BSL feed.

Order	Family	Genus	Relative Abundance (%) ^a^	Number of Isolates
Bacillales	*Bacillaceae*	*Bacillus*	15.8	4
Bacillales	*Bacillaceae*	*Lysinibacillus*	2.8	3
Bacillales	*Planococcaceae*	*Rummeliibacillus*	0.073	2
Micrococcales	*Micrococcaceae*	*Glutamicibacter*	0.0036	1

^a^ Mean relative abundance of the genus in the gut microbiota of the BSL catfish group.

**Table 3:** Acquired ARGs detected in the intestinal microbiomes of groups BSL and CONT ^a^.

	Acquired ARGs Detected with the Coverage (%)								
Antibiotic Class	AMN	AMN	TRI	FOS	MAC	QNL	SUL	TET	TET
Fish Group/*ARG*	*aadA9*	*aph(3′)-Ia*	*dfrG*	*fosB*	*lnu(C)*	*qnrD1*	*sul1*	*tetA(P)*	*tetB(P)*
BSL	74.5%		60.6%	48.2%	100%	52.2%	63.5%	100%	99.3%
CONT		100%			100%			100%	99.3%

^a^ ARGs detected at ≥40% coverage and ≥80% identity with the reference gene. Abbreviations for antibiotic classes are AMN, aminoglycoside; TRI, trimethoprim; FOS, fosfomycin; MAC, macrolide; QNL, quinolone; SUL, sulfonamide; TET, tetracycline.

## Data Availability

The original contributions presented in this study are included in the article/Supplementary Materials. Further inquiries can be directed to the corresponding authors. NGS datasets analyzed in this study were submitted to the NCBI Sequence Read Archive (SRA) with accession PRJNA1042795, and to the NCBI Genomes database under project PRJNA1006294.

## Supplementary Materials

The following supporting information can be downloaded at: https://www.mdpi.com/article/10.3390/ani15091338/s1, Supplementary Table S1: Formulation, proximate composition, gross energy and amino-acid and fatty-acid profiles of the control and experimental diets used in the digestibility experiment; Supplementary Table S2: African catfish intestinal contents samples collected for analyses of their microbiota composition; Supplementary Table S3: Known or putative chitinase genes of bacterial isolates cultured from the BSL diet; Supplementary Table S4. Known or putative chitinase genes detected in the intestinal microbiomes of the BSL catfish group; Table S5. Known or putative chitinase genes detected in the intestinal microbiomes of the CONT catfish group; Supplementary Table S6: Acquired ARGs identified in selected studies of African catfish (*Clarias gariepinus*) or Channel catfish (*Ictalurus punctatus*). Supplementary Figure S1. Significant genus-level changes between groups START and CONT. Supplementary Figure S2. Intestinal metagenomic contigs of the BSL catfish group.
