# Supplementary material for: Effects of Short-Term Feeding with Diets Containing Insect Meal on the Gut Microbiota of African Catfish Hybrids
Source: Animals (Basel). 2025 May 6;15(9):1338. doi: 10.3390/ani15091338 (PMC12071142; doi:10.3390/ani15091338)
Supplement: Supplementary file 1 [file animals-15-01338-s001.zip › animals-3589960-supplementary.pdf]

SUPPLEMENTARY MATERIALS

Experimental diets

The diets for the digestibility study contained defatted black soldier fly larvae (BSL), yellow mealworm (MW) or full fat blue bottle fly larvae (BBF) meals, respectively, in 70:30 ratio between the control (CONT) basal diet and the tested insect meals. The indirect method for digestibility study was performed using 0.1% yttrium oxide as an inert marker [4].

Supplementary Table S1. Formulation (g kg^{-1}), proximate composition (%, wet weight), gross energy (MJ kg^{-1}, wet weight) amino acid and fatty acid profile (w %) of the control and experimental diets used in the digestibility experiment [4].

	CONT diet	BSL diet	MW diet	BBF diet
**Ingredients**	**Formulation (g kg^{-1})**			
Fish meal[1]	399	280	280	280
Winter wheat [2]	330	230	230	230
Soybean protein concentrate[3]	130	91	91	91
Corn gluten[4]	110	77	77	77
Vitamin/mineral premix[5]	30	21	21	21
Insect meal[6]	0	300	300	300
Yttrium-oxide[7]	1	1	1	1
	**Proximate Composition % (mean ± SD)**			
Dry Matter	95.69 ± 0.02	95.30 ± 0.06	96.87 ± 0.06	96.05 ± 0.01
Crude Protein*	47.13 ± 0.14	50.10 ± 1.00	53.14 ± 0.75	48.96 ± 0.60
Crude Fat	5.80 ± 0.12	8.50 ± 0.05	5.90 ± 0.45	12.10 ± 0.79
Crude Fiber	1.43 ± 0.07	4.05 ± 0.03	1.70 ± 0.01	3.55 ± 0.17
Crude Ash	9.01 ± 0.04	8.33 ± 0.04	8.34 ± 0.04	7.66 ± 0.01
Phosphorus	1.03 ± 0.01	0.89 ± 0.01	0.85 ± 0.02	0.94 ± 0.03
Calcium	1.79 ± 0.01	1.98 ± 0.00	1.35 ± 0.00	1.40 ± 0.07
Gross energy (MJ kg^{-1})	18.63 ± 0.06	19.92 ± 0.04	19.23 ± 0.02	20.66 ± 0.04
Acid Detergent Fiber (ADF)	2.13 ± 0.33	6.11 ± 0.76	10.06 ± 0.47	6.34 ± 0.12
Chitin[8]	0.13 ± 0.05	4.91 ± 0.54	2.94 ± 0.56	3.06 ± 0.37
	**Essential Amino Acid (EAA)%**			
Arginine (ARG)	2.59	2.80	3.01	2.79
Histidine (HIS)	0.97	1.12	1.01	1.28
Isoleucine (ILE)	1.82	2.01	1.83	1.96
Leucine (LEU)	4.01	3.98	4.22	3.91
Lysine (LYS)	4.35	4.61	4.45	5.29
Methionine (MET)	0.89	0.94	0.93	1.01
Threonine (THR)	1.88	2.05	2.02	2.09
Phenylalanine (PHE)	2.21	2.36	2.42	2.82
Tryptophan (TRP)	3.07	3.07	3.64	3.38
Valine (VAL)	2.19	2.70	2.46	2.46
**Σ EAA**	**23.99**	**25.63**	**25.99**	**27.00**
**Σ AA**	**42.88**	**45.49**	**46.16**	**47.22**

FOOTNOTES TO SUPPLEMENTARY TABLE S1

[1] 999 LT Fish meal, Triple Nine Fish Protein A/S, Esbjerg, Denmark.

[2] Supplied from a local feed mill, Novi Sad, Serbia.

[3] Tradkon SPC500-P, Sojaprotein, Bečej, Serbia.

[4] Starch Industry, Jabuka DOO, Pančevo, Serbia.

[5] Ravago Chemicals (Feketić, Serbia).

[6] BSL: Black soldier fly larvae supplied by Agroloop Ltd.; MW: yellow mealworm from Berg and Schmidt Pte. Ltd Singapore; BBF: blue bottle fly produced by Csali Hungary Ltd.

[7] Alfa Aesar, Thermo Fisher (Kandel) GmbH, Karlsruhe, Germany.

[8] Chitin % = ash free ADF % - ADF protein % following the method of Marono et al., 2015.

* Crude Protein was calculated by applying a nitrogen to protein conversion factor of Kp=6.25.

Supplementary Table S2. African catfish intestinal content samples collected for analyses of their microbiota composition.

Nr.	Code of animal	Full length (cm)	Body weight (g)	Sample type	Treatment groups A	Treatment groups B
1	AH8	29.3	215.4	intestine content	START	START
2	AH9	32.6	296.2	intestine content	START	START
3	AH10	30.7	258.2	intestine content	START	START
4	AH11	35.7	363.8	intestine content	START	START
5	AH12	34	353.6	intestine content	START	START
6	AH13	32.6	306.4	intestine content	START	START
7	AH15	35.8	436.6	intestine content	BBF[a]	IM[d]
8	AH16	36	443.8	intestine content	BBF	IM
9	AH17	39.5	599.4	intestine content	BBF	IM
10	AH18	37.1	436.6	intestine content	BBF	IM
11	AH20	34.7	409.2	intestine content	BBF	IM
12	AH21	37.1	463	intestine content	BBF	IM
13	AH22	38.9	530	intestine content	BBF	IM
14	AH25	38.8	521.8	intestine content	BSL[b]	IM
15	AH26	36.3	431.4	intestine content	BSL	IM
16	AH27	36.9	430.2	intestine content	BSL	IM
17	AH28	33.5	306.2	intestine content	BSL	IM
18	AH29	36.1	401.4	intestine content	BSL	IM
19	AH30	37.2	449.6	intestine content	BSL	IM
20	AH31	37.7	531	intestine content	BSL	IM
21	AH35	38.5	502.6	intestine content	MW[c]	IM
22	AH36	39.1	509.2	intestine content	MW	IM
23	AH37	35.2	384.4	intestine content	MW	IM
24	AH38	35.1	360.4	intestine content	MW	IM
25	AH39	39.9	560.2	intestine content	MW	IM
26	AH40	36.9	466	intestine content	MW	IM
27	AH41	36	416.8	intestine content	MW	IM
28	AH42	38.9	543.6	intestine content	CONT	CONT
29	AH43	39.9	562.4	intestine content	CONT	CONT
30	AH44	41.1	642.4	intestine content	CONT	CONT
31	AH45	38	433.2	intestine content	CONT	CONT
32	AH46	38.2	502	intestine content	CONT	CONT
33	AH47	36.3	389.6	intestine content	CONT	CONT
34	AH48	34.7	402.6	intestine content	CONT	CONT
35	AH49	33.5	340.2	intestine content	CONT	CONT
36	AH50	37.1	410.8	intestine content	CONT	CONT

[a] BBF: blue bottle fly (*Calliphora vicina*) larvae meal group.
[b] BSL: black soldier fly (*Hermetia illucens*) larvae meal group.
[c] MW: yellow mealworm (*Tenebrio molitor*) meal group.
[d] IM: the insect meal group IM was defined as groups BBF, BSL and MW pooled into one group.

Supplementary Table S3. Known or putative chitinase genes of bacterial isolates cultured from the BSL diet[a]

Genome sequenced isolate [a]	Locus tag	Encoded protein	Accession of BLASTX hit	Identical residues	Identity (%)
Bacillus sp. BSL6	RCJ96_05930	Chitinase	WP_074608217	360/360	100
Bacillus sp. BSL6	RCJ96_21370	Chitinase	EEL19093	669/674	99
Lysinibacillus sp. BSL11	RCJ95_05255	GH18 protein[b]	WP_221681264	347/348	99
Glutamicibacter sp. BSL13	RCJ92_01695	GH18 protein[b]	TLK48683	541/542	99

[a] WGS sequencing data for bacterial isolates cultured from the BSL diet.
[b] Glycosyl hydrolase family 18 putative chitinase-like protein [55].

Supplementary Table S4. Known or putative chitinase genes detected in the intestinal metagenome of the BSL catfish group [a]

Contig assemb-ler	Contig length (bp)	NCBI BLASTX protein hit	Accession of BLASTX hit	Identical residues	Identity (%)
MEGAHIT	458	Chitinase (*Plesiomonas shigelloides*)	EON87728	107/109	98
MEGAHIT	341	Chitin binding protein (*P. shigelloides*)	WP_256855523	105/105	100
MEGAHIT	375	Chitinase (*Bacillota bacterium*)	HAX72310	72/96	75
MEGAHIT	1154	Chitinase (*Flavimobilis rhizosphaerae*)	WP_192279536	170/180	94
MEGAHIT	690	Chitinase (*Sanguibacter* sp.)	QIK84567	54/66	82
PEAR	266	GH18 protein (*Lysinibacillus* spp.) [b]	TKI71875	88/88	100
PEAR	211	Chitin deacetylase (*Bacillus cereus*)	ANJ04699.1	70/70	100
PEAR	180	Chitinase D (*Bacillus cereus* AH820)	ACK91888	60/60	100
PEAR	157	Chitinase (*Glutamicibacter* sp.)	WP_138178394	52/52	100
PEAR	115	GH18 protein (*Glutamicibacter* sp.) [b]	WP_176484090	37/38	97
PEAR	111	Chitinase (*Glutamicibacter* sp.)	WP_138178394	36/36	100
PEAR	98	Chitinase (*Lysinibacillus* sp.)	KAB0445980	31/32	97
PEAR	98	Chitinase (*Bacillus cereus*)	TKI81715.1	32/32	100
PEAR	150	Chitinase (*Bacillus amyloliquefaciens*)	AIU96338.1	50/50	100
PEAR	97	Chitinase (*Bacillus cereus*)	ACO50698	31/31	100
PEAR	105	Chitinase C (*Bacillus cereus*)	EEL02456	34/34	100
PEAR	98	Chitinase (*Bacillus mycoides*)	OSX87431.1	32/32	100
PEAR	150	Chitinase (*Bacillus* sp.)	QUS93875	50/50	100

[a] Shotgun metagenomic data for the intestinal content of the BSL group catfishes AH25 and AH31
[b] Glycosyl hydrolase family 18 (GH18) putative chitinase-like protein [55].

Supplementary Table S5. Known or putative chitinase genes detected in the intestinal metagenome of the CONT catfish group [a]

Contig assembler	Contig length (bp)	NCBI BLASTX protein hit	Accession of BLASTX hit	Identical residues	Identity (%)
PEAR	133	Chitinase (*P. shigelloides*)	KAB7661545.1	29/31	94
PEAR	85	Chitin binding protein (*P. shigelloides*)	WP_256855523	28/28	100

[a] Shotgun metagenomic data for the intestinal content of the CONT group catfishes AH43 and AH44

Supplementrary Table S6. Acquired ARGs identified in selected studies of African catfish (AC) or Channel catfish (CC)

Antibiotic Class	Species	Country	Acquired ARGs	References
Quinolone	AC	Hungary	*qnrD1*	this study
		Egypt	*qnrA, qnrB, qnrS*	[86]
		Germany	*qnrS*	[89]
Tetracycline	AC	Hungary	*tetA(P), tetB(P)*	this study
		Egypt	*tetA*	[85, 86]
		Malaysia	*tetA, tetE*	[88]
	CC	United States	*tetA, tetB, tetC, tetD, tetE,tetM, tetR, tet(34), tet(35)*	[90-94]
		China	*tetA, tetB, tetC, tetD, tetE, tetG, tetJ, tetL, tetM, tetO, tetQ, tetR, tetS, tetPA, tetPB, tetU, tetV, tetX, tet(32), tet(34), tet(36)*	[95]
Phenicol	CC	United States	*catB1, catB2, catB7, cmlA1, floR*	[90, 94]
		China	*cmlA1, floR*	[95]
Macrolide	AC	Hungary	*lnuC*	this study
	CC	United States	*msrE, mphE,vatF*	[90, 94]
		China	*ermA, ermB, ermF, ermK, ermT, ermX, erm(34), ereA, lnuB, lnuC, mefA, mphA, mphB, msrA, oleC, vatB, vatC*	[95]
Aminoglycoside	AC	Hungary	*ant(3'')-Ia (aadA9), aph(3')-Ia*	this study
		Egypt	*aadA1*	[86]
		Malaysia	*strA-strB, aadA*	[88]
	CC	United States	*aac(2')-IIa, aph(3'')-Ib, aph(4)-Ia, aph(6)-Id, aadA1, aadA2, aadA5, aadA7*	[93, 94]
		China	*aadA*	[95]
Colistin	CC	United States	*mcr-3, mcr-7*	[90]
Sulphonamide	AC	Hungary	*sul1*	this study
		Germany	*sul1*	[89]
		Egypt	*sul1*	[86]
		Malaysia	*sul1*	[88]
	CC	United States	*sul1, sul2*	[94]
		China	*sul1, sul2, sulA/folP*	[95]
Trimethoprim	AC	Hungary	*dfrG*	this study
	CC	United States	*dfrA3, dfrA12, dfrA16, dfrA17*	[90, 93, 94]
		China	*dfrA1, dfrA12*	[95]
Fosfomycin	AC	Hungary	*fosB*	this study
β-lactam	AC	Egypt	bla_{TEM}, bla_{CTX-M}	[85]
			bla_{TEM}, bla_{CTX-M}	[86]
		Malaysia	bla_{TEM}, bla_{SHV}	[88]
		Germany	$bla_{CTX-M-32}$, bla_{TEM}	[89]
	CC	United States	bla_{EC}, bla_{TEM}, bla_{CTX-M}, bla_{SHV}, *imiS, ampS*	[87, 90, 94]
		China	bla_{TEM}, bla_{ACC-1}, bla_{CMY}, bla_{CTX-M}, bla_{GES}, bla_{L1}, bla_{OXA}, bla_{PAO}, bla_{PSE}, bla_{ROB}, bla_{SFO}, bla_{SHV}, bla_{VEB}, bla_{VIM}, bla_{Z}, *ampC, cfiA, fox5*, bla_{NDM}, *penA*, bla_{SHV}, bla_{Z}	[95]

SUPPLEMENTARY FIGURES

Supplementary Figure S1. Significant genus-level changes between groups START and CONT

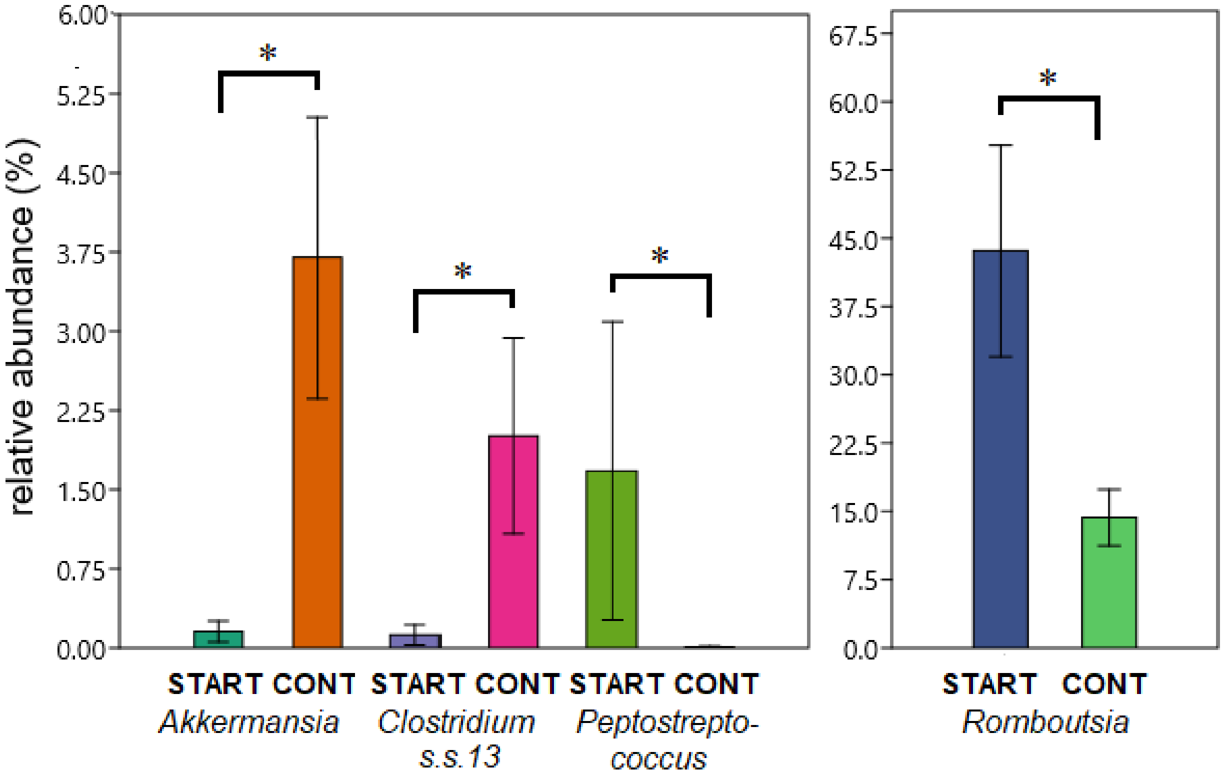

Supplementary Figure S1. Significant differences in relative abundances (± SE) of the genera *Akkermansia* (p=0.046), *Clostridium sensu stricto 13* (p=0.046), *Peptostreptococcus* (p=0.028), and *Romboutsia* (p=0.028) between groups START and CONT. *Difference significant at the p<0.05 level.

Supplementary Figure S2. Intestinal metagenomic contigs of the BSL catfish group [a]

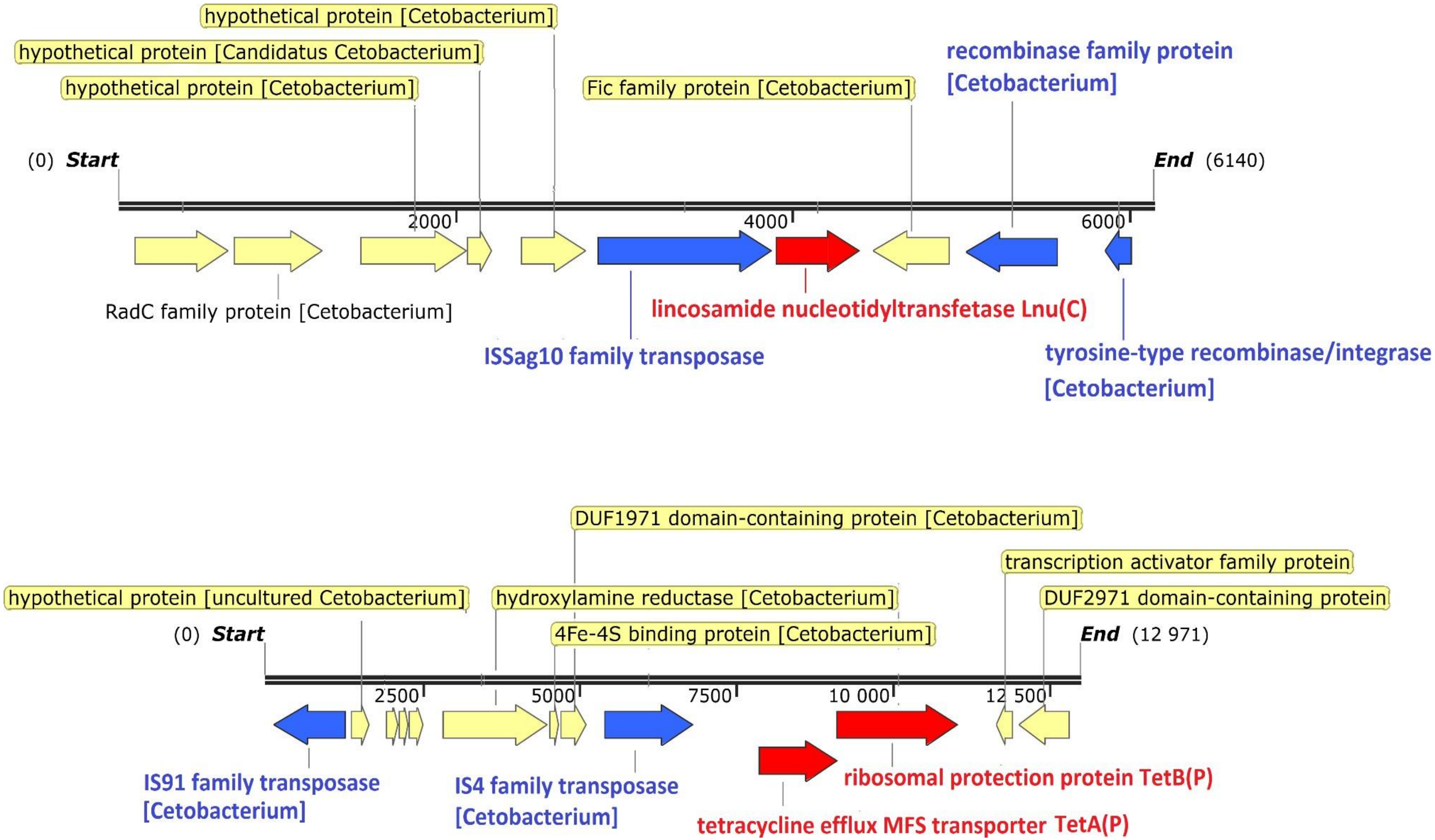

[a] For further details on these intestinal metagenomic contigs harboring *lnu(C)* or *tetA(P)* and *tetB(P)* genes, respectively, see Table 3 and text.
